# The rationale for using low-molecular weight heparin in the therapy of symptomatic COVID-19 patients

**DOI:** 10.1515/med-2021-0374

**Published:** 2022-01-24

**Authors:** Edyta Reichman-Warmusz, Oliwia Warmusz, Romuald Wojnicz

**Affiliations:** Department of Histology and Cell Pathology in Zabrze, School of Medicine with the Division of Dentistry, Medical University of Silesia in Katowice, Jordana 19, 41-808 Zabrze, Poland; Silesian Centre for Heart Disease in Zabrze, Zabrze, Poland; Department of Histology and Cell Pathology in Zabrze, School of Medicine with the Division of Dentistry, Medical University of Silesia in Katowice, Zabrze, Poland

**Keywords:** COVID-19, inflammation, microvascular coagulapathy, anticoagulation, low-molecular weight heparin

## Abstract

Accumulated evidence suggest that the adverse outcome of severe coronavirus disease 2019 (COVID-19) is closely related to prothrombotic microvascular pathology with a high risk of venous thromboembolism. Furthermore, the first observational studies indicated that adjunct therapy with low-molecular weight heparin (LMWH) was associated with lower mortality in this cohort of patients. However, the timing of starting LMWH and the dose remain controversial in COVID-19 patients. Considering the above, the aim of this study was to reveal the rationale for using LMWH in the therapy of symptomatic COVID-19 patients based on experimental and clinical studies on LMWH in inflammatory settings with special consideration given to randomized trials.

## 1 Introduction

So far, acute respiratory syndrome and multiorgan failure caused by severe acute respiratory syndrome coronavirus-2 (SARS-CoV-2) infection have been related to the fatal consequences of the coronavirus disease 2019 (COVID-19) [1,2]. Despite the fact that the pathogenesis of multiorgan injury due to SARS-CoV-2 is far from elucidated, the problem of microcirculation dysfunction combined with thrombotic microangiopathy is constantly raised [3,4,5]. In addition, prothrombotic microvascular pathology with a high risk of venous thromboembolism is suggested to be responsible for the adverse outcome of COVID-19 patients [6,7]. Of note, microcirculation abnormalities found in patients with COVID-19 have been also reported in severe heart failure (HF) and myocardial infarction complicated by cardiogenic shock with a higher risk of death in these conditions [8,9]. It is worth emphasizing that a significantly higher mortality rate has been reported in patients infected by SARS-CoV-2 who are elderly, diabetic, hypertensive, or have any other cardiovascular disease [10]. These pathological conditions are characterized by the presence of small vessel disease with endothelial dysfunction [11,12]. Considered together, it is not surprising that the cardiovascular system is often affected by the SARS-CoV-2 infection [13,14]. And indeed, as reported by Shi et al., among 62 of 671 patients who died, more than 75% patients were more likely to have myocardial injury [15].

Several mechanisms have been proposed that may lead to cardiac microcirculatory dysfunction in the course of viral infections: (1) a persistent inflammatory response, which can beget local thrombosis, (2) neurohormonal activation including elevated levels of catecholamines, and (3) tissue hypoperfusion with subsequent development of oxidative stress [16,17,18]. Considered together, the question is whether microcirculation dysfunction may be a therapeutic target in symptomatic COVID-19 patients.

Anticoagulant therapy with low-molecular weight heparin (LMWH) reduced the incidence of thrombotic complications without an increase in bleeding events and was associated with lower mortality in seriously ill COVID-19 patients [19,20]. However, as mentioned before, the use of heparin in terms of timing and dose is controversial [21,22]. Therefore, the following crucial questions remain to be addressed: (1) When should LMWH therapy be introduced? (2) Which dose should be recommended – prophylactic or therapeutic? (3) How long should the patients be treated during hospitalization or in outpatient settings? Bearing in mind the above, the aim of this study was to reveal the rationale for using LMWH in the therapy of symptomatic COVID-19 patients considering the results of adjunct therapy with LMWH in cardiac pathology related to microvascular dysfunction and inflammatory conditions.

### 1.1 Lesson from *in vitro* and animal studies on heparin used in inflammatory settings

Since 1964, a number of studies have reported the *in vitro* inhibition of viruses using heparin [23,24]. Further studies focused on the potential mechanisms of heparin action in antiviral therapy [25,26]. These studies reported that heparin could significantly reduce the accumulation of leukocytes in the inflammatory settings, for example, in the skin and lungs [27,28]. Moreover, *in vitro* experiments showed that heparin could inhibit proinflammatory cytokine gene expression by lipopolysaccharide-stimulated human mononuclear cells [29,30]. Furthermore, the direct comparison between unfractionated and LMWHs revealed similar and significant anti-inflammatory properties of both of them, and the inhibition of nuclear factor kB (NF-κB) activation is one of the potential mechanisms of these properties [31]. In addition to the inhibition of NF-κB, decreased expression of serum tumor necrosis factor and circulating p38 mitogen-activated protein kinase levels by heparin in acute lung injury in rabbits was also reported [32]. Heparin can also bind to many adhesion molecules expressed during inflammation, including selectins and integrins [33]. Apart from the above mechanisms, heparin can also influence tissue remodeling processes by inhibiting heparanase, the enzyme that results in the cleavage of heparan sulfate HS-chains, thereby affecting the structure and function of the extracellular matrix [34].

### 1.2 Lesson from the clinical studies on LMWH with special attention to randomized trials on HF patients

The first observational studies indicated that COVID-19 patients treated with LMWH had a better survival rate [1,20]. Therefore, there is no doubt that the use of anticoagulant therapy with LMWH is of paramount importance and currently recommended thromboprophylaxis in COVID-19 patients [35]. However, its efficacy should be validated in such a cohort of patients with special attention to time for starting LMWH therapy and the dose used [21,22].

Searching for answers to these questions, it is worth analyzing two randomized trials on LMWH including HF patients in whom inflammatory settings with procoagulant conditions were evident, and the viruses were involved in its development [36,37,38,39].

The first randomized trial by De Lorenzo et al. aimed to determine whether a short-term therapy with LMWH was safe in patients with HF [40]. Furthermore, their single-center randomized study was performed to test the hypothesis that a prophylactic dose of LMWH (bemiparin sodium) would modify a hypercoagulable state in HF patients. One hundred patients with HF were randomized to bemiparin sodium (3,500 IU/daily subcutaneously) or placebo for 5 days. In the bemiparin group, a significant decrease in D-dimer, factor VII, and thrombin–antithrombin complex was found. However, a significant increase in protein C was reported at the same time as compared with placebo.

**Figure 1:**
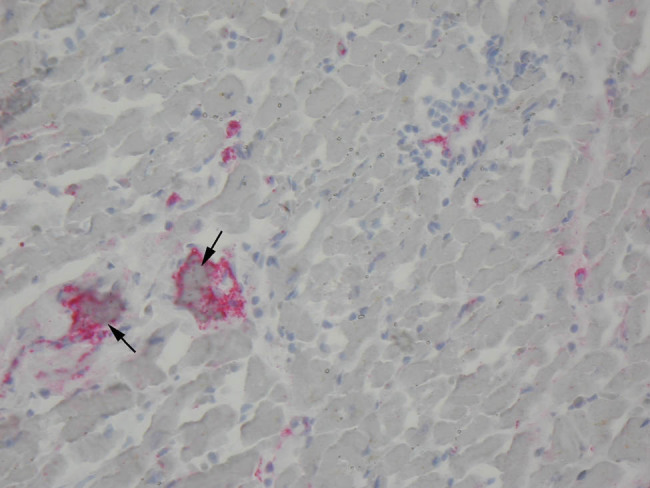
Endomyocardial biopsy sections of a patient with chronic HF secondary to inflammatory dilated cardiomyopathy treated with therapeutic doses of LMWH for 10 weeks. Immunohistochemistry staining of von Willebrand Factor (vWF). Baseline biopsy shows occluded microvessels by thrombi (arrows) with a strong expression of vWF in the settings of cell inflammatory infiltration.

As opposed to the trial by De Lorenzo et al., our study was mainly related to the assessment of the efficacy of therapeutic doses of LMWH (enoxaparin) in a long-term therapy (3 months) and long-term (12 months) maintenance of potential improvement in a similar cohort of HF patients. The first randomized trial with therapeutic doses of LMWH in HF patients was published in 2006 [41]. One-hundred and two patients were randomized to receive either enoxaparin for 3 months (1.5 mg/kg/day s.c.) or conventional treatment for HF alone. At a 12 month follow-up, the thrombin–antithrombin complex decreased significantly in the enoxaparin-treated patients as compared with the baseline data. Similarly, plasma N-terminal brain natriuretic peptide (NT-proBNP) concentrations significantly decreased in LMWH-treated patients (Figure 1).

**Figure 2:**
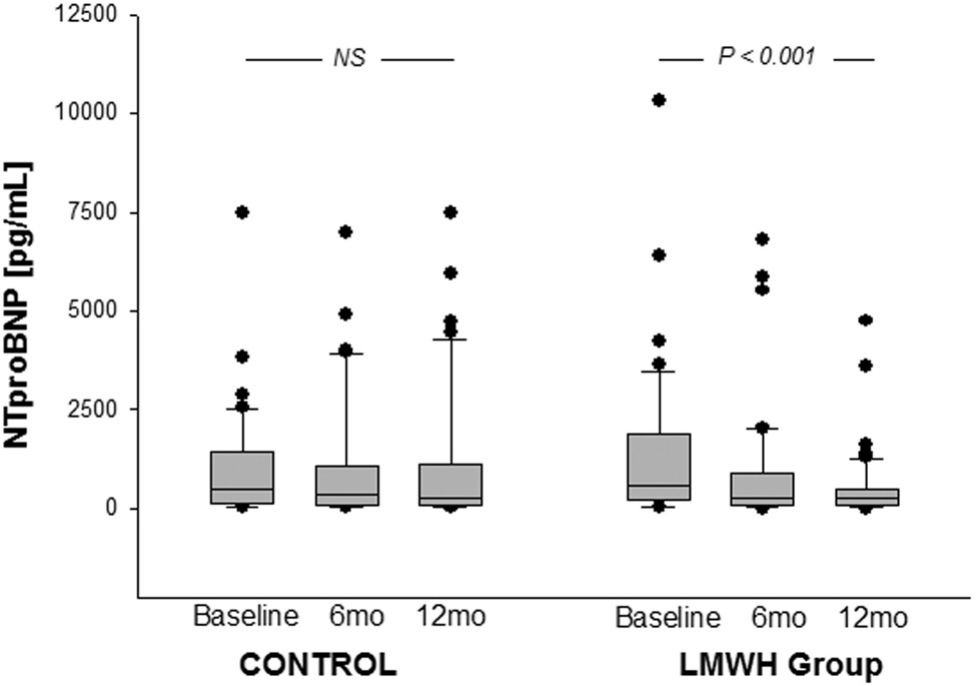
Plasma NT-proBNP concentrations in patients randomized to LMWH or placebo. NT-proBNP decreased significantly in the LMWH-treated patients (Data adopted from ref. [41]).

Apart from that, the long-term LMWH therapy resulted in a sustained beneficial effect on cardiac function without deleterious effects on major cardiac events in such a cohort of patients. In several patients, right ventricular endomyocardial biopsy was performed, which suggested the effectiveness of LMWH in the therapy of inflammatory conditions (Figure 2). Our finding of decreased thrombin–antithrombin complex levels after LMWH therapy is in line with the study of De Lorenzo et al. [40].

Based on these observations and their extrapolation to COVID-19 patients, it seems to be rational to introduce therapeutic doses of LMWH immediately after the first occurrence of symptoms and follow such an approach for at least 10 weeks in outpatient settings.

## 2 Conclusion

In most cases, the SARS-CoV-2 infection affects highly vascularized organs, including the lungs, heart, and kidney. In addition, a significantly higher mortality is reported in patients infected with SARS-CoV-2 who are elderly, diabetic, hypertensive, or have any other cardiovascular disease. Importantly, all these pathologies are characterized by their dysfunctional microcirculation with hypercoagulability. As a result, the SARS-CoV-2 infection facilitates inflammatory microvascular dysfunction and diffuse intravascular coagulation in the already existing pathology. Anticoagulant therapy with LMWH was associated with lower mortality and reduced the incidence of thrombotic complications without an increase in bleeding events in seriously ill COVID-19 patients. These findings seem to indicate that the use of LMWH can improve the microcirculatory function in patients with pre-existing endothelial dysfunction. It may be suggested that adjunct long-term therapeutic doses of LMWH may be beneficial for COVID-19 patients without deleterious effects in a long-term therapy. We believe that the extrapolation of the results of these two trials to severely ill COVID-19 patients might be useful in the decision-making process. In our opinion, patients hospitalized due to COVID-19 should receive therapeutic doses of LMWH immediately after hospitalization, which should be continued for a long time in the outpatient setting (based on our experience of at least 10 weeks).
